# Protected Health Information filter (Philter): accurately and securely de-identifying free-text clinical notes

**DOI:** 10.1038/s41746-020-0258-y

**Published:** 2020-04-14

**Authors:** Beau Norgeot, Kathleen Muenzen, Thomas A. Peterson, Xuancheng Fan, Benjamin S. Glicksberg, Gundolf Schenk, Eugenia Rutenberg, Boris Oskotsky, Marina Sirota, Jinoos Yazdany, Gabriela Schmajuk, Dana Ludwig, Theodore Goldstein, Atul J. Butte

**Affiliations:** 1 Bakar Computational Health Sciences Institute, University of California, San Francisco, San Francisco, CA USA; 2 Division of Rheumatology, Department of Medicine, University of California, San Francisco, San Francisco, CA USA; 3 San Francisco Veterans Affairs Medical Center, San Francisco, CA USA; 4 Center for Data-Driven Insights and Innovation, University of California Health, Oakland, CA USA

**Keywords:** Medical research, Health care

## Abstract

There is a great and growing need to ascertain what exactly is the state of a patient, in terms of disease progression, actual care practices, pathology, adverse events, and much more, beyond the paucity of data available in structured medical record data. Ascertaining these harder-to-reach data elements is now critical for the accurate phenotyping of complex traits, detection of adverse outcomes, efficacy of off-label drug use, and longitudinal patient surveillance. Clinical notes often contain the most detailed and relevant digital information about individual patients, the nuances of their diseases, the treatment strategies selected by physicians, and the resulting outcomes. However, notes remain largely unused for research because they contain Protected Health Information (PHI), which is synonymous with individually identifying data. Previous clinical note de-identification approaches have been rigid and still too inaccurate to see any substantial real-world use, primarily because they have been trained with too small medical text corpora. To build a new de-identification tool, we created the largest manually annotated clinical note corpus for PHI and develop a customizable open-source de-identification software called Philter (“Protected Health Information filter”). Here we describe the design and evaluation of Philter, and show how it offers substantial real-world improvements over prior methods.

## Introduction

Structured electronic health records (EHRs) fields, primarily comprises elements such as high-level demographics and billing codes (ICD), are currently the most utilized in determining the state of a patient, in terms of clinical care details or disease state. Many of these fields are often used in clinical research, and are now starting to be used to determine human phenotypes^1^ for genome-wide association studies, and can be used to facilitate automated improvements of healthcare decision-making^2^. However, in many cases this information is not detailed enough to provide appropriate insights. For example, Breast Imaging Reporting and Data System (BI-RADS) scores, which provide a radiologist-assigned assessment of breast cancer status-based mammogram can only be found in radiology reports at UCSF. Similarly, physician documentation of patient symptoms can only be found in the clinical notes at our hospital. Additionally, procedural, diagnostic, and medication billing coding are often incomplete, inconsistent, subjective, and inaccurate (often due to the needs of billing prioritizing over the needs of science), and this could even lead to false insights^3,4^. Clinical notes often contain the richest and most relevant information available about disease phenotypes, treatments, and outcomes, as well as the clinical decision-making process. This written medical narrative frequently captures patient experience and event ordering timelines. To date, there have been many studies that have successfully used data from clinical notes for discoveries, including detection of drug adverse outcomes^5^, identification of off-label drug use^6^, surveillance of disease states^7^, and identification of clinical concept relatedness^8^.

With nearly the entire United States healthcare system now adopting EHRs, but with most of the actual clinical details captured in these free-text notes, transforming information contained within clinician notes into a computable resource is essential for medical research and improving patient care. However, clinical notes contain legally Protected Health Information (PHI), which prevent their use in most research applications.

Removal of PHI from clinical notes is a challenging task because the potential number of words that could be PHI are limitless. There are many different methods for recording and formatting patient note data across the health system landscape, and each health system serves a distinct patient population, resulting in differences in the distribution of types of PHI across health systems^9^ and the probability that a given word is PHI or a medical term (e.g.: “MA”).

The current state-of-the-art in de-identification systems still have real-world weaknesses because there are only a small number of corpora openly available for algorithm development and testing^10–14^. Priorities around de-identification software performance in recent years have been driven largely by de-identification competitions, most notably the Integrating Biology and the Bedside (i2b2) competitions in 2006 and 2014, which have emphasized a balanced approach of information retention and patient privacy, instead of national guidelines (https://www.hhs.gov/hipaa/for-professionals/privacy/special-topics/de-identification/index.html), which focus exclusively on privacy. It is clear that real-world performance is generally still below the threshold of compliance regulations for removing PHI, resulting in a lack of broader use of these tools to de-identify notes for research^9,15,16^. Every piece of PHI not identified and removed represents a potential violation of patient privacy and also a potentially expensive lawsuit. Even at 95% recall (i.e., percent of PHI removed), the amount PHI still remaining across millions of clinical notes would be staggering.

With an incredibly diverse patient population being treated at the University of California, San Francisco (UCSF), yielding over 70 million clinical notes collected within our Electronic Health Records (EHRs), we required an efficient, accurate, and secure method for removing PHI from notes in order to make these data usable by researchers while minimizing the risk of PHI exposure. We developed a privacy-centric approach to removing PHI from free-text clinical notes using both rule-based and statistical natural language processing (NLP) approaches. The algorithm utilizes an overlapping pipeline of methods that are state-of-the-art in each application including: pattern matching, statistical modeling, blacklists, and whitelists. We built this software tool as a self-contained system that could be deployed on any major computing platform and can operate without an internet connection, allowing it to be run in secure environments.

We have called this algorithm Philter (Protected Health Information filter). In this work, we describe the engineering of Philter and its evaluation against other systems. As we have discovered most existing tools in this field do not have actual open-source availability, we have released Philter as open source code, and envision tens of thousands of health systems finding it useful.

## Results

### Study design

The Inter-Rater Reliability, for PHI vs. Safe tokens, between first and second pass annotators in the UCSF corpus was greater than 99.99%, with the second annotator identifying an average of 39 additional PHI tokens and converting an average of 21 tokens from PHI to Safe per 500 notes.

We compared overall recall and precision and per-PHI-category. Recall across the three algorithms (Physionet, Scrubber, and Philter) on two corpora; the 2000 note UCSF test corpus mentioned above and the publicly available 514 note 2014 i2b2 test corpus.

Primary and Secondary result metrics on both corpora are displayed in Table 1, with precision listed as a reference. On the UCSF test corpus: Physionet had a recall of 85.10% and an F2 of 86.15%, Scrubber had a recall of 95.30% and an F2 of 91.59%, and Philter had a recall of 99.46% and an F2 of 94.36%. On the 2014 i2b2 test corpus: Physionet had a recall of 69.84% and an F2 of 73.05%, Scrubber had a recall of 87.80% and an F2 of 85.22%, and Philter had a recall of 99.92% and an F2 of 94.77%.

**Table 1:** Performance comparison of tools and corpora.

	UCSF			I2B2		
	*P*	*R*	F2	*P*	*R*	F2
PHIlter	78.28	99.46	94.36	78.58	99.92	94.77
Physionet	90.62	85.10	86.15	89.49	69.84	73.05
Scrubber	79.24	95.30	91.59	76.26	87.80	85.22

Performance comparison of tools and corpora.

*P* precision, *R* recall.

Philter also outperformed both of the other algorithms for each category of PHI on both corpora, in addition to having the highest overall recall (Tables 2, 3).

**Table 2:** Remaining PHI analysis by tool, UCSF test corpus.

PHI category	Instances of PHI remaining (PHIlter)	Instances of PHI remaining (Physionet)	Instances of PHI remaining (Scrubber)
Age ≥ 90	0	0	0
Patient_Vehicle_or_Device_Id	0	18	0
Patient_Account_Number	0	35	4
Patient_Medical_Record_Id	0	445	0
Patient_Social_Security_Number	0	0	6
Patient_Phone_Fax	0	0	1
Patient_Initials	2	120	132
Patient_Name_or_Family_Member_Name	6	211	93
Patient_Address	7	25	16
Patient_Unique_ID	20	442	34
Email	0	1	1
URL_IP	4	20	153
Date	7	257	269
Provider_Certificate_or_License	0	276	99
Provider_Name	12	546	90
Provider_Initials	12	236	217
Provider_Address_or_Location	43	1597	210
Provider_Phone_Fax	45	49	43

PHI counts for PHIlter, Physionet and Scrubber performance on the UCSF corpus. Instances of PHI represent single tokens within the span of multiple or single-token items of PHI.

**Table 3:** Remaining PHI analysis by tool, I2B2 corpus.

PHI category	Instances of PHI remaining (PHIlter)	Instances of PHI remaining (Physionet)	Instances of PHI remaining (Scrubber)
Age	0	1	0
Device	0	6	0
Medical record	0	524	18
Patient	2	154	92
Date	0	4590	1587
Fax	0	2	0
Phone	0	31	67
Zip	0	3	1
Username	1	92	92
Street	2	27	21
Location-other	2	9	12
Idnum	2	297	206
City	2	14	52
Doctor	5	197	186

PHI counts for PHIlter, Physionet and Scrubber performance on the I2B2 corpus.

#### Sensitivity analysis

Distribution of PHI and Philter Recall by Category.

The raw count of PHI varied noticeably between the two corpora, but Philter’s recall consistently generalized across the categories for each corpus (Supplementary Tables 4, 5).

Results of additional sensitivity analyses regarding the precision errors caused by each element of the algorithm pipeline (Supplementary Table 6) and the impact of partial PHI removal (Supplementary Tables 7, 8) and can be found in the Supplement.

The amount of real (wall-clock) time necessary to run 500 notes as a single process was 323 s. The amount of real time necessary to simultaneously process 20 batches of 500 notes, 10,000 notes total, was 401 s.

## Discussion

In this study, we developed an algorithm, Philter, that utilizes an overlapping pipeline of multiple state-of-the-art methods and compared it to the two strongest real-world competitors on the basis of recall. Philter demonstrated the highest overall recall on both corpora, had the highest recall in each category of PHI on both corpora, and generalized well between the corpora. Philter’s recall on the 2014 i2b2 test corpus is the highest reported in the literature. A key design decision was the use of rules to separate PHI from Safe words while using a statistical method to improve precision. The overall size of the UCSF corpus at 4500 manually annotated notes is the largest in the world that we are aware of. Likewise, the UCSF test corpus, at 2000 notes, is the largest corpus to be tested and reported in the literature.

With more EHR systems being deployed across the world, there is still an incredible need for text processing tools, and de-identification is a key utility that can enable many readers and programmers to access those notes in a safer manner. While challenges and competitions have been run for nearly 10 years, there is still a pragmatic need for safe, efficient, open-source de-identification tools.

The field has been dominated by two separate approaches to designing de-identification algorithms. The first uses a rule-based system to detect PHI, while the second approach uses statistics to assign probabilities of PHI to words. Rule-based systems primarily use regular expressions and/or blacklists of words to tag PHI. Statistical methods employ machine learning, traditionally Conditional Random Fields and increasingly Recurrent Neural Networks, to learn patterns based on words and their context. Rule-based systems typically have better recall, while statistical methods typically have better precision. Rule-based systems are inherently predictable allowing their success and failures to be anticipated. Statistical systems are much faster to build; however, they are often difficult to interpret and performance on new data is more unpredictable. For example, the organizers of the 2006 i2b2 challenge discovered that the best performing algorithm in the competition, which utilized a statistical approach, suffered serious failures when de-identifying notes that came from the same hospital but were not drawn from the competition corpus^17^.

The sparsity of available notes for de-identification system development and testing has provided a tremendous challenge to developing robust de-identification approaches because the nature of PHI contained within a note may differ significantly depending on the hospital or department they were generated from. Ferrandez et al.^9^ demonstrated this by showing different proportions of categories of PHI distribution between the VHA, i2b2, and the Swedish Stockholm corpora. For example, Provider Names comprised only 9% of the overall PHI in the VA corpus, but were 19% of the PHI in i2b2, while there were no occurrences of Provider Names in the Stockholm corpus. Conversely, Patient Names make up only 4 and 5% of the VA and i2b2 PHI, respectively, but over 20% of the Stockholm corpus. ID Numbers were barely present in the VA corpus, totaling less than half of 1% of the PHI, but were responsible for >24% of the PHI in the i2b2 corpus.

Between the systems selected as comparators for this study, the Physionet tool is the oldest and most “proven”; it has great precision but does not effectively remove PHI. Scrubber is a newer software and the designers traded precision to get much improved recall. Unfortunately, neither of these approaches can be easily modified. Since PHI varies widely from corpus to corpus and the needs of those performing de-identification are diverse, the lack of customizability of these tools presents real-world usability challenges.

The NLM Scrubber software assumes that words appearing frequently in public documents are unlikely to be PHI, and although this assumption appears reasonable, it is not justifiable given our own findings. As mentioned above, we found over 16,000 names in the census and Social Security data that were either common English words or medical terms. This may explain the 20x difference between Scrubber and Philter in the number of patient name tokens that remained after filtering.

In addition to outperforming the comparators selected for this study, Philter sets new state-of-the-art recall results on the 2014 i2b2 corpus. The challenge winner, the Nottingham system, had a recall of 96.29 (micro-averaged, token-wise, Health Insurance Portability and Accountability Act (HIPAA) category)^12^. Philter also demonstrates higher recall than the results reported for the more modern deep-learning based de-identification systems (Dernoncourt et al.^18^ i2b2 recall 97.38; Lui et al.^19^ recall 93.8). Interestingly, the only publically available de-identification system used in the aforementioned competition, MITRE’s MIST tool^20^, faired quite poorly (HIPAA token recall of 80.05) even when supplemented with the well regarded Stanford NER tagger and pre-trained on an additional private corpus from Kaiser.

It is fair to note that the i2b2 Challenge systems and the deep-learning systems mentioned in this manuscript attempted to maximize F1 rather than recall. While we believe that this is a flawed approach within the de-identification community (considering recall is the primary concern from a patient privacy standpoint), we acknowledge that tuning these systems to maximize PHI removal could potentially improve their recall performance.

As mentioned above, the part of speech (POS) tagger portion of the pipeline was the most problematic element from a precision perspective. Despite having lower recall and being subject to several statistical system challenges, such as lack of transparency and great risk of poor generalization to new corpora, we are excited by the very high precision of the deep-learning approaches previously referenced^18,19^. We can imagine replacing the current NLTK POS tagger in the Philter pipeline with a deep-learning version of the same.

Despite Philter’s strong performance, with recall values equal to or greater than 99.5%, recall still was not perfect. The portions of PHI that were not identified were edge cases around existing patterns. For example, there were six total tokens that were missed for patient names in the UCSF test corpus. These tokens actually came from one single patient, whose name was six tokens long. The six token name appeared twice in one note, and each time Philter successfully removed three of the names, likely making the actual patient’s name difficult or impossible to re-identify. The solution to this and similar problems are almost trivially easy to fix but they underscore the need to test de-identification systems on very large and diverse corpora to continually discover and refine edge cases.

The statistical portion of the pipeline was the most problematic from a precision perspective. The POS tagger frequently confused capitalized words, either at the beginning of sentences or all-capital words within sentences, as proper nouns. We found a very high overlap between common English words and medical terms (See, Whitelist) with names taken from the Census and Social Security. Precisely 16,095 names were found to be either medical terms or common English words. Therefore, an incorrect POS tag of NNP frequently resulted in a false positive.

The decision not to include institution-specific information, such as a map between patient names and note identification numbers, could be considered a limitation. At the time of development, we chose not to include such information for numerous reasons. First, our lists of patient names are messy (it was not uncommon for drug names to appear as patient names in our databases). Second, even after rigorous initial cleaning, our patient name lists only detected 80% of name PHI within the corpus. This is in part due to the fact that patient family member names frequently appear within notes and in part due to misspellings of names. Third, relying on the use of inside data would not produce an algorithm that was generalizable out of the box. We believe that patient name-to-note maps could make a small but valuable addition to the pipeline and we envision placing it prior to the Names Blacklist steps. However, at the time of this writing, despite extensive development, we still are not ready to incorporate them. If we find that doing so improves performance in the future, we will provide the steps necessary to reproduce our process at other institutions on our github README.

In summary, Philter providers state-of-the-art de-identification performance while retaining the majority of relevant medical information. We envision that PHI removal can be further optimized using a crowd-sourcing approach with lots of exposure to many hospitals and notes. For this reason, we have made Philter open-source and highly customizable. We believe the system is capable of 100% recall with enough exposure and community involvement. The simple to use software will accept any text file as input, is fully modular to allow the community to improve the algorithm or adapt it to each users’ specific needs, easy to evaluate, and executable in a secure environment. The software comes pre-configured, as the pipeline described in this manuscript, to produce the de-identification results that most closely follow HIPAA Safe Harbor guidelines.

To our knowledge there is no current standard for quantify the cost of information lost to false positives during de-identification. We believe that this is in large part due to the fact that the relevance of each non-PHI word is tightly coupled to specific clinical or research questions. Cost of information loss due to de-identification could be addressed by a research project that considered multiple common and important tasks for de-identified clinical notes. Those tasks could be performed using two different sets of data as input; (a) the raw notes and (b) the de-identified notes output by Philter. Performance results could then be compared to quantify “de-identification cost” for each task. Additionally, we will monitor Philter’s github repository to incorporate improvements from additional users.

## Methods

### Study design

The UCSF Committee on Human Research approved our study protocol [study # 16-20784]. The IRB waived the requirement for individual Research HIPAA Authorization for all subjects. The UCSF IRB determined the use or disclosure of the requested information did not adversely affect the rights and welfare of the individuals and involves no more than a minimal risk to their privacy. The UCSF IRB determined the requested waiver of informed consent was acceptable.

To create the UCSF corpus of clinical notes, 4500 notes were randomly selected from over 70 million notes from all departments at UCSF by assigning a hash identity to each note ID, randomly permuting the order of the hashed ID, then randomly selecting 4500 hashed note IDs. Words were then manually annotated for PHI-categories by one of our three trained annotators. The annotators used Multi-document Annotation Environment (MAE)^21^. The MAE tool was configured with PHI elements following the HIPAA Safe Harbor guidelines with a couple of additional categories to identify provider information (Supplementary Table S1). 4500 notes were annotated twice, with a second annotator reviewing and correcting the mark-up of the first annotator and Inter-Rater Reliability, which provides the percent agreement between annotators, was calculated. When in doubt, annotators chose the more conservative option, for example marking an unclear name as belonging to a patient vs. a physician. We generated a distribution of the randomly sampled notes and found >100 note categories, note types, departments of origin, and provider specialties. We randomly assigned 2500 notes to use for the development of a new de-identification algorithm (see Supplementary Table 2 for a distribution of the departments represented) and 2000 notes to test algorithm performance (Supplementary Table 3).

The i2b2 2014 de-identification challenge test corpus consists of 514 notes and was downloaded on 18 July, 2017^10,11^. However, annotations of words as either safe or PHI within this corpus do not exactly follow the HIPAA guidelines for Safe Harbor, specifically in regards to locations and dates^22^. We, therefore, changed the annotations for words from the following categories: years in isolation, seasons (e.g., winter, spring), days of the week, single letters with no adjacent content, country names and ages under 90 from PHI to safe. The i2b2 2014 corpus replaced real PHI with surrogates. In a few instances, the surrogate values are for patient identification numbers were unrealistic, being four digits or less. These were removed.

The categories of PHI, the values of PHI, and the context surrounding PHI within a note can change drastically between types of notes, between departments within a health system, and between different health systems. In contrast to this, we believe that words that are not PHI have considerably less variability. Therefore, we started with an approach of identifying words that are not likely to be PHI. Approaches to identify words that are likely to be PHI were then incorporated into the algorithm for additional security and precision.

To optimize ease of use and modularity, while ensuring that the complete algorithm performs as expected, the pipeline is controlled by a simple text configuration file in the JavaScript Object Notation (JSON) format. We store the position of each character in memory so that tokens identified as PHI may be replaced with an obfuscated token of exactly the same length (e.g.,: “John Smith” becomes “**** *****”). Therefore, the original structure of the note is perfectly preserved, with the exception that asterisks in the original note are replaced with spaces. The priority with which a token is marked as PHI or safe is dictated by the order of processes in the configuration file and is entirely customizable. We built an evaluation script that automatically compares de-identified notes to annotated gold-standards at the character level to quantify global and PHI category-specific performance.

At the beginning of the pipeline, a custom script tokenizes individual words within each note by separating them on whitespace and symbols (i.e., -, /, #, &, periods, etc). Next, short phrases that have a high probability of not being PHI are identified using pattern matching with a custom library of 133 “safe” regular expressions. Then, a custom library of 171 regular expressions is used to identify predictable PHI entities such as salutations, emails, phone numbers, dates of birth, social security numbers, and postal codes. In both cases, the regular expressions search for specific words, phrases, and/or numbers and utilize the immediate context surrounding each word to identify matches. For example, if a number appears adjacent to the word “age” or “years old”, that number is interpreted as an age and is PHI if it is greater than or equal to ninety, as per HIPAA guidelines for Safe Harbor methods. On the other hand, a number referring to dosage (e.g., 50 mg) is not interpreted as PHI.

At this stage, the Python NLTK module is used to tag each word with a POS to address the challenge of dealing with words that could be either safe or PHI, using statistical modeling to determine the structure of each sentence and document. For example, the word “White” in the context of “White fluid found at…” is an adjective and therefore safe, while “Patient John White presents with…” is a proper noun and is PHI.

We assembled a blacklist of names using last names occurring 100 or more times in the 2010 U.S. census, and first names occurring five or more times for each year of birth between 1879–2017 from the U.S. Social Security website. To minimize occurrences of names that are also common words (i.e., new, walks, knee, home, child, etc.) in the blacklist, we removed a total of 855 words from the blacklist that were the greatest contributors to precision errors during training (complete documentation of blacklist creation is available on the public github repository). All names added to the final blacklist were tokenized on whitespace and symbols, and converted to lowercase. The blacklist was separated into a first names blacklist and a last names blacklist, and the two lists were incorporated into the full pipeline in succession. During the blacklist stage of PHI-searching, if a token is in at least one of the blacklists and is labeled as a proper noun by NLTK (e.g., POS tag = NNP), it is marked as PHI, tokens not in the lists are left unmarked.

Next, an additional name removal step is implemented using a combination of regular expression and blacklist matching. We created a custom library of four regular expressions that search for common last name patterns in clinical notes (e.g., Jane Doe or Doe, Jane), and potential names are marked as PHI if an adjacent token was previously marked as PHI by a blacklist.

At this point, the pipeline employs a safety mechanism to catch PHI that occurs in unexpected formats, such as previously unseen names, words with incorrect POS tags, or misspellings. This is accomplished by identifying previously unlabeled (label = PHI/Safe) tokens that are most likely not PHI. This is accomplished using a custom whitelist of ~195,000 tokens comprises medical terms and codes extracted from common medical word banks and ontologies (e.g., UMLS, SNOMED, MeSH, etc.), common medical abbreviations, the 20,000 most common English words and an additional list of common English verbs with varied tenses. All Social Security and 2010 Census names were removed from the whitelist, and some common English and medical words were then added back to the whitelist to maintain acceptable precision measurements (Complete whitelist documentation can be found on the github repository). All tokens that have not already been categorized as PHI or Safe by an earlier portion of the pipeline, with the exception of tokens with numeric POS tags, are passed through the whitelist.

A final active filtering process is used to identify patient and provider initials. We created a single regular expression that searches for initials patterns in clinical notes (e.g., Doe, J. or Jane S. Doe), and these regex matches are marked as PHI if one or more adjacent tokens were previously marked as PHI by a blacklist.

**Fig. 1:**
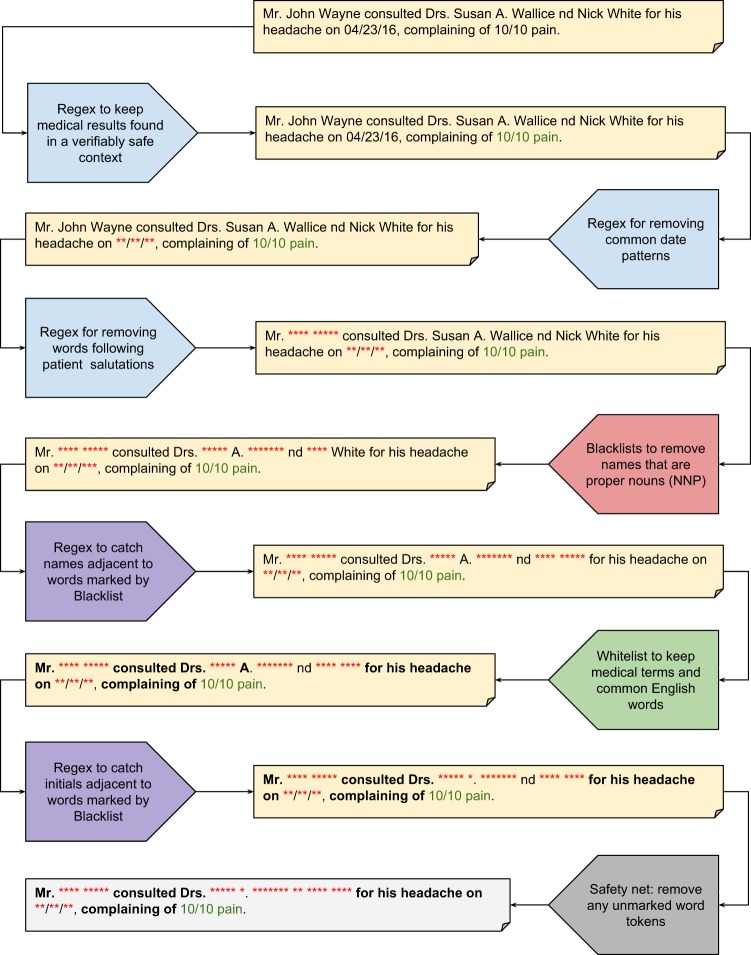
Algorithm Pipeline. A conceptual overview of the philter pipeline and process.

At the conclusion of the pipeline a token can have one of three possible labels: marked for exclusion, marked for inclusion, or unmarked. To maximize patient privacy, only words marked for inclusion are retained (Fig. 1).

Two-thousand five-hundred notes in the UCSF development corpus were used to develop the optimal Philter algorithm. Each portion of the pipeline, as well as the overall ordering of the pipeline, was modified to obtain the greatest overall performance metrics. Examples include changes to regular expression patterns, the tokens present in the White and Black lists, and the POS tags used to match against the lists. Optimization was done iteratively, developing against 500 notes at a time from the development set, testing against the next 500 notes in the development set, then repeating, growing the size of the development set by the previous 500 notes each time.

Ferrandez et al.^9^, performed a head-to-head comparison of multiple de-identification systems on multiple corpora, which revealed that the PhysioNet de-identification tool^11^, had the best out-of-the-box performance. To identify PHI, the PhysioNet algorithm uses a combination of regular expressions and three types of lookup dictionaries (known names of patients and hospital staff, generic names of people and locations, and common words along with UMLS terms considered by their team unlikely to be PHI).

We selected the PhysioNet de-identification tool as the strongest comparator that met our criteria of open-source software that could be deployed entirely behind a firewall and downloaded the source code from PhysioNet’s^14^ website (https://www.PhysioNet.org/physiotools/deid/) on 12 February, 2017.

The National Library of Medicine’s Scrubber tool, first published in 2013^23^ takes the approach of maximizing recall and valuing real-world generalization over public challenge competition results. It has been continually revised and improved since its initial creation and investigators have even launched a trial with updates as recent as 2018. The tool makes use of other public tools, including Apache’s cTAKES^24^ and UIMA projects^25^, to compare the likelihood of words being PHI based on their relative frequency of appearance in public documents such as medical journals and LOINC codes to private physician notes under the reasonable assumption that words that appear in public documents are unlikely to be PHI. We selected the NLM Scrubber tool as our second comparator and downloaded the most recent version (v.18.0928) from the NLM website (https://scrubber.nlm.nih.gov/files/). Unfortunately, NLM Scrubber software does not maintain the original character alignment of scrubbed notes and comes with no method to automatically evaluate its performance against annotated notes. We had to design an evaluation script for this software and have made the script available to the community on our GitHub repository.

If PHI is allowed through a de-identification system, that yields a recall error, in that the PHI was not found. If safe words are obfuscated, that yields a precision error, in that extra text was unnecessarily removed. Since preventing exposure of PHI is our highest priority, we wanted to devise a system that minimized recall errors, even at the expense of greater precision errors.

Each PHI word that evades detection increases the risk of patient re-identification. Therefore, we evaluate performance at the word-level. In this analysis, we count as True Positives (TP) those PHI words that were correctly labeled as PHI while the False Positives (FP) are non-PHI words that were incorrectly labeled as PHI. Likewise, True Negatives (TN) are non-PHI words correctly labeled as non-PHI while False Negatives (FN) are PHI words incorrectly labeled as non-PHI.

Since we chose to optimize our method to maximally maintain patient privacy, we chose recall as our primary measure of performance (Eq. 1), which represents the portion of PHI words that were identified correctly:

1 $${\mathrm{Recall}} = {\mathrm{TP}}/{\mathrm{TP}} + {\mathrm{FN}}$$

However, de-identified clinical notes only have value if they retain as much non-PHI information as possible. Thus, we also measure precision (Eq. 2), which represents the portion of filtered words that were non-PHI:

2 $${\mathrm{Precision}} = {\mathrm{TP}}/{\mathrm{TP}} + {\mathrm{FP}}$$

To account for precision, we selected the F2 score (Eq. 3) as our secondary performance measure, which is a weighted average of recall and precision that values recall twice as much as precision:

3 $${\mathrm{F2}} = 5 \ast {\mathrm{Precision}} \ast {\mathrm{Recall}}/\left( {4 \ast {\mathrm{Precision}}} \right) + {\mathrm{Recall}}$$

In addition to Recall and F2 performance, we were interested in we were interested in the distribution of PHI across each category of PHI along with the number of TPs and FPs resulting from the best de-identification tool.

**Fig. 2:**
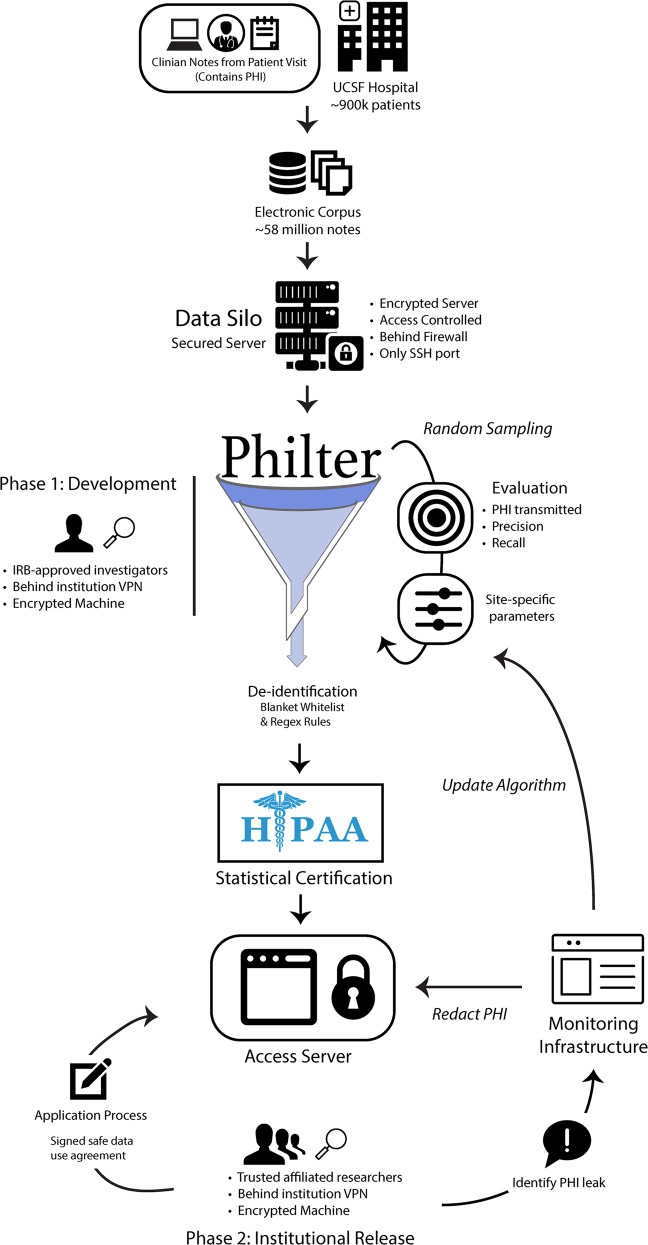
Ecosystem. The compute environment and system used for the development and validation of Philter.

Figure 2 outlines the environment we designed to build and run Philter on clinical notes while ensuring security of the original notes and providing a framework for reporting PHI that was not filtered by the algorithm.

To ensure security, clinical notes were kept on a server with an encrypted drive protected behind an institutional firewall and through access-controlled VPN at all times from initial software development through institutional release. Access to the server was only permitted via password-protected Secure Shell (SSH) protocol from points inside the VPN, and only from devices which themselves had encrypted stores or hard drives. The raw clinical notes were loaded onto the server through a Clarity-level text document extraction from UCSF’s Epic EHR system.

We calculated the run time of our pipeline using batches of 500 notes on a 32 core Linux machine with 16 GB of RAM using the native Linux Time function, “time”, to estimate the feasibility of running Philter at a large scale. We conducted two experiments. First, a single batch of 500 notes, with a total size of 2.2 Mb, was run as single process and timed. Second, 20 batches of the 500 notes were run simultaneously as multiple processes and timed.

The Philter package is written in machine-portable Python. The package can be installed via PIP, the Python package installer, and the source code along with detailed design descriptions, as well as installation and use instructions can be obtained through the public repository open-sourced, under an MIT License (https://github.com/BCHSI/philter-ucsf).

### Reporting summary

Further information on research design is available in the Nature Research Reporting Summary linked to this article.

## Supplementary information

Supplementary Information

Reporting Summary

## Data Availability

The Philter package is written in machine-portable Python. The package can be installed via PIP, the Python package installer, and the source code along with detailed design descriptions, as well as installation and use instructions can be obtained through the public repository open-sourced, under an MIT License (https://github.com/BCHSI/philter-ucsf).

## Data availability

The i2b2 data that support the findings of this study are available from i2b2 but restrictions apply to the availability of these data, which require signed safe usage and research-only. Data from UCSF are not available at this time as they have not been legally certified as being De-Identified, however, this process is underway and the data may be available by the time of publication by contacting the authors. Requesters identity as researchers will need to be confirmed, safe usage guarantees will need to be signed, and other restrictions may apply.
