# Supplementary material for: Protected Health Information filter (Philter): accurately and securely de-identifying free-text clinical notes
Source: NPJ Digit Med. 2020 Apr 14;3:57. doi: 10.1038/s41746-020-0258-y (PMC7156708; doi:10.1038/s41746-020-0258-y)
Supplement: Supplementary file 1 — Supplementary Information [file 41746_2020_258_MOESM1_ESM.pdf]

Supplemental Background

Motivation

Initially, our plan was to identify a pre-existing de-identification system that we could use for this task. The number of open-source publicly available de-identification software systems is very small. We began the search for such a system by examining the HIPAA-defined, token-based, recall results of the i2b2 2014 de-identification challenge[1] [1]. Unfortunately, the top performing entry, Nottingham system [2], was specifically fine-tuned for both the i2b2 dataset as well as the i2b2 evaluation script (using a post-processing script to modify tokens to maximize scoring), potentially limiting its generalization and resulting in over-optimistic assessment of performance. Additionally, the Nottingham system is not publicly available for use. Interestingly, the only publicly available de-identification algorithm that was used in the competition, MITRE's MIST tool[3], faired quite poorly (HIPAA token recall of .805) even when supplemented with the well regarded Stanford NER tagger and pre-trained on an additional private corpus from Kaiser.

A wider literature review of post-i2b2 challenge identified a couple of potentially promising candidates that used Deep Recurrent Neural Networks and reported results on the i2b2 2014 corpus for comparison[4,5]. However, the Lui et al. system is not publicly available in any form, and while the Dernoncourt et al. team have made available a Named Entity Recognition tagger based on their work, the de-identification system reported in their paper is not available.

Existing De-Identification Corpora

There are a very small number of public corpora that have been labeled for PHI and are available to develop or test de-identification algorithms. The Informatics for Integrating Biology and the Bedside (i2b2) program, released a corpus of 889 discharge summaries as part of a challenge in 2006 to evaluate state-of-the-art systems for automatically targeting and removing PHI [6]. In 2008, PhysioNet released a corpus of 2,434 nursing notes that they used to build a software de-identification tool [7,8]. In 2014, i2b2 released another corpus as part of a new challenge consisting of 1,304 longitudinal clinical narratives derived from 295 hand-selected diabetic patients at risk for coronary artery disease [1,9].

Supplemental Methods

UCSF Corpora

Supplemental Table 1: PHI Categories

PHI Categories
**Age >= 90**
**Patient_Vehicle_or_Device_Id**
**Patient_Account_Number**
**Patient_Medical_Record_Id**
**Patient_Social_Security_Number**
**Patient_Initials**
**Patient_Name_or_Family_Member_Name**
**Patient_Address**
**Patient_Unique_ID**

**Email**
**URL_IP**
**Date**
**Phone_Fax**
**Provider_Certificate_or_License**
**Provider_Name**
**Provider_Initials**
**Provider_Address_or_Location**

Supplemental Table 2: Distribution of 2500 training notes Across Departments

**Department_Specialty**	**Count**
**Gastroenterology**	**233**
**Obstetrics**	**225**
**Radiology**	**181**
**General Internal Medicine**	**177**
**Pulmonology**	**161**
**Pulmonary Function and Bronchoscopy**	**133**
**Ophthalmology**	**128**
**Obstetrics and Gynecology**	**121**
**Emergency Medicine**	**117**
**Family Medicine**	**103**
**Dermatology**	**82**
**Cardiology**	**75**
**Reproductive Endocrinology and Infertility**	**60**
**Kidney Transplantation**	**54**
**Endocrinology and Metabolism**	**51**
**Urologic Oncology**	**50**
**Hepatology**	**48**
**Primary Care**	**46**
**General Pediatrics**	**43**
**Neurology**	**40**
**Orthopedic Surgery**	**39**
**Liver Transplant**	**38**
**Neurosurgery**	**38**
**Anesthesiology**	**35**

Pediatric Gastroenterology	35
Otolaryngology, Head and Neck Surgery	33
Radiology MR	30
Rheumatology	27
Radiology CT	26
Hematology and Oncology	25
Urology	25
Lung Transplant	20
Breast Care - Cancer Center	19
Pediatric Nephrology	19
Psychiatry	19
Allergy and Immunology	15
Interventional Radiology	15
Pediatric Cardiology	15
Geriatric Medicine	13
Lab	13
Nephrology	13
Pediatric Endocrinology	13
Pediatric Neurology	13
Gastrointestinal Oncology	12
Physical Therapy	12
Dysplasia	11
HIV Program	10
Infusion and Transfusion	10
Pediatric Oncology	10
Pediatric Rheumatology	10
Gynecologic Oncology	9
Prenatal Diagnosis	9
Pain Medicine	8
Radiation Oncology	8
Anticoagulation	6
Heart Transplant	6
Nuclear Medicine	6

Pathology	6
Adolescent Medicine	5
Employee Health Services	5
Pediatric Hematology	5
Pediatric Otolaryngology, Head and Neck Surgery	5
Thoracic Oncology	5
General Surgery	4
Genetics - Cancer Center	4
Investigational Therapy	4
Optometry	4
Pediatric Pulmonology	4
Plastic Surgery	4
Executive Health	3
Home Health Services	3
Orthotics	3
Pediatric Immunology	3
Pediatric Urology	3
Sleep Medicine	3
Audiology	2
Colorectal Surgery	2
Endocrine Surgery	2
Orthopedic Surgical Oncology	2
Pediatric Anesthesiology	2
Pediatric Orthopedic Surgery	2
Pediatric Physical Medicine and Rehabilitation	2
Pediatric Surgery	2
Respiratory Therapy	2
STOR Immunizations Converted	2
Surgical Oncology	2
Thoracic Surgery	2

**Vascular Lab**	**2**
**Cardiothoracic Surgery**	**1**
**Clinical Research**	**1**
**Craniofacial Anomalies**	**1**
**Diabetes Services**	**1**
**Hospice and Palliative Medicine**	**1**
**Hospital Medicine**	**1**
**Infectious Diseases**	**1**
**Interpreting Services**	**1**
**Melanoma**	**1**
**Pediatric Bone Marrow Transplant**	**1**
**Pediatric Infectious Disease**	**1**
**Pediatric Infusion and Transfusion**	**1**
**Pediatric Occupational Therapy**	**1**
**Pediatric Pulmonary Function**	**1**
**Social Services**	**1**
**Support Service - Cancer Center**	**1**
**Vascular Surgery**	**1**

Supplemental Table 2: Department Specialties are uniquely coded by UCSF and retrieved as meta-data from the notes. Many notes do not contain this specific meta-data field. Only non-null values for department_specialty are reported here.

Supplemental Table 3: Distribution of Testing Notes Across Departments

Department_Specialty	Count

Obstetrics	95
Radiology	73
Pulmonology	71
General Internal Medicine	70
Gastroenterology	69
Ophthalmology	66
Pulmonary Function and Bronchoscopy	64
Emergency Medicine	60
Endocrinology and Metabolism	51
Obstetrics and Gynecology	51
Family Medicine	50
Kidney Transplantation	38
Cardiology	34
Dermatology	30
Hepatology	27
Primary Care	26
Reproductive Endocrinology and Infertility	26
General Pediatrics	22
Liver Transplant	20
Neurosurgery	19
Pediatric Gastroenterology	19
Urologic Oncology	18
Hematology and Oncology	17
Neurology	17
Orthopedic Surgery	17
Radiology CT	15
Otolaryngology, Head and Neck Surgery	13
Radiology MR	13
Urology	13
Rheumatology	12
Anesthesiology	11
Gastrointestinal Oncology	10
Interventional	10

Radiology	
Breast Care - Cancer Center	9
Lung Transplant	9
Nephrology	8
Pediatric Endocrinology	8
Geriatric Medicine	7
Lab	5
Pediatric Nephrology	5
Anticoagulation	4
Dysplasia	4
Executive Health	4
Pediatric Cardiology	4
Pediatric Rheumatology	4
Psychiatry	4
Radiation Oncology	4
General Surgery	3
Interpreting Services	3
Investigational Therapy	3
Neuro-Interventional Radiology	3
Pathology	3
Pediatric Neurology	3
Respiratory Therapy	3
Thoracic Oncology	3
Adolescent Medicine	2
Allergy and Immunology	2
Employee Health Services	2
Gynecologic Oncology	2
Heart Transplant	2
HIV Program	2
Infusion and Transfusion	2
Orthopedic Surgical Oncology	2
Pediatric Pulmonology	2
Prenatal Diagnosis	2
Surgical Oncology	2

**Audiology**	**1**
**Endocrine Surgery**	**1**
**Endocrinology**	**1**
**Hospital Medicine**	**1**
**Integrative Medicine**	**1**
**Melanoma**	**1**
**Nuclear Medicine**	**1**
**Optometry**	**1**
**Pain Medicine**	**1**
**Pediatric Diabetes**	**1**
**Pediatric Hematology**	**1**
**Pediatric Oncology**	**1**
**Pediatric Orthopedic Surgery**	**1**
**Physical Therapy**	**1**
**Plastic Surgery**	**1**
**Sleep Medicine**	**1**
**Social Services**	**1**
**Symptom Management**	**1**

Supplemental Table 3: Department Specialties are uniquely coded by UCSF and retrieved as meta-data from the notes. Many notes do not contain this specific meta-data field. Only non-null values for department_specialty are reported here.

Sensitivity Analysis

In addition to Recall, F2 performance, and our primary sensitivity analysis, we were interested in two additional sensitivity analysis. First, we were interested in determining the impact of partial de-identification successes, specifically, were there instances where only a portion of the PHI was removed that made the changed remaining associated tokens from PHI to safe. An example would be obscuring part of a date (eg: 1/1/2018 → */*/2018) or most of a name (eg: John A Smith → **** A *****). Second, while not emphasizing Precision as a de-identification metric, we wanted to catalog which elements of the Philter pipeline were the greatest contributors to precision errors to better anticipate which types of non-PHI words were most likely to be erroneously removed.

Supplemental Results

Supplemental Sensitivity Analysis One: What PHI Actually Remains after de-identification
Even when de-identification failed to completely remove an entire PHI entity, approximately 20% of the time it removed enough of the entity to make it no longer recognizable as PHI

Supplemental Sensitivity Analysis Two: Precision Errors
The portions of the pipeline that search for names were the most significant contributors to precision errors.

Supplemental Table 4. Recognizable PHI Analysis (PHIlter, UCSF Test Corpus)

PHI Category	Recognizable PHI

**Age >= 90**	0
**Patient_Vehicle_or_Device_Id**	0
**Patient_Account_Number**	0
**Patient_Medical_Record_Id**	0
**Patient_Social_Security_Number**	0
**Patient_Phone_Fax**	0
**Patient_Initials**	0
**Patient_Name_or_Family_Member_Name**	6
**Patient_Address**	4
**Patient_Unique_ID**	11
**Email**	0
**URL_IP**	0
**Date**	6
**Provider_Certificate_or_License**	0
**Provider_Name**	11
**Provider_Initials**	6
**Provider_Address_or_Location**	40
**Provider_Phone_Fax**	45

Supplemental Table 4. Recognizable PHI counts for PHIlter performance on the UCSF corpus. We defined "recognizable PHI" as any annotated identifier that was not PHI according to HIPAA after surrounding PHI was removed. There were 158 total FNs for Philter on the UCSF corpus initially, with 129 recognizable as PHI by human analysis after de-identification. Refer to the "Not Recognizable PHI" column in Supplemental Table 3 for detailed information on criteria used for determining recognizable PHI.

Supplemental Table 5. Recognizable PHI Analysis (PHIlter, I2B2 Corpus)

PHI Category	Recognizable PHI
**AGE**	0
**DEVICE**	0
**MEDICALRECORD**	0
**PATIENT**	2
**DATE**	0
**FAX**	0
**PHONE**	0
**ZIP**	0
**USERNAME**	0
**STREET**	2
**LOCATION-OTHER**	2
**IDNUM**	0
**CITY**	2

**DOCTOR**	4

Supplemental Table 5. Recognizable PHI counts for PHIlter performance on the i2b2 test corpus. There were 16 total FNs for Philter on the UCSF corpus initially, with 12 recognizable as PHI by human analysis after de-identification.

Supplemental Table 6. False Positive Count by PHIlter Configuration File Element on the UCSF corpus

Filter	False Positive Count
Last Names Blacklist (lastnames_minus_fps.json)	1830
Whitelist	1725
First Names Blacklist (firstnames_minus_fps.json)	1236
'filters/regex_context/names_regex_context3.txt'	649
'filters/regex_context/initials.txt'	508
'filters/regex/dates/mm_yy_transformed.txt'	366
'filters/regex/addresses/hospital2.txt'	356
'filters/regex/dates/mm_dd_transformed.txt'	301
'filters/regex_context/names_regex_context2.txt'	252
'filters/regex/addresses/in_city_transformed.txt'	242
'filters/regex/ucsf_regex/ucsf_neighborhoods.txt'	226
'filters/regex/contact/xxx_xxx_xxxx.txt'	191
'filters/regex/salutations/post_salutations_2chars.txt'	172
'filters/regex/dates/dd_mm_transformed.txt'	161
'filters/regex/dates/month_name_transformed.txt'	108
'filters/regex/dates/mm_dd_yy_transformed.txt'	102
'filters/regex/salutations/pre_salutations_2chars.txt'	101

Supplemental Table 6. Each row name corresponds directly a file process within the pipeline and its relative location on the software filepath. False positive (FP) counts for PHIlter configuration file elements with FP counts >=100. Because multiple filters matched some FPs, FP counts do not reflect total number of FPs generated by PHIlter, but rather the total number of times each filter matched any FP.

Supplemental Table 7: UCSF corpus

PHI Category	TPs	FNs	Recall
**Age >= 90**	11	0	100.00%

**Patient_Vehicle_or_Device_Id**	550	0	100.00%
**Patient_Account_Number**	35	0	100.00%
**Patient_Medical_Record_Id**	471	0	100.00%
**Patient_Social_Security_Number**	30	0	100.00%
**Patient_Initials**	721	2	99.72%
**Patient_Name_or_Family_Member_Name**	1579	6	99.62%
**Patient_Address**	3996	7	99.83%
**Patient_Unique_ID**	652	20	97.02%
**Email**	120	0	100.00%
**URL_IP**	468	4	99.15%
**Date**	13396	7	99.95%
**Phone_Fax**	1469	45	97.03%
**Provider_Certificate_or_License**	369	0	100.00%
**Provider_Name**	5045	12	99.76%
**Provider_Initials**	721	12	98.36%
**Provider_Address_or_Location**	3998	43	98.94%

Supplemental Table 7 TP/FN counts and recall per PHI category for PHIlter performance on the UCSF test corpus. The following annotated PHI categories were not considered PHI for performance evaluation purposes, and not included in performance analysis:.

Supplemental Table 8. Overall Recall Per PHI Category (PHIlter, I2B2 Test Corpus)

PHI Category	TPs	FNs	Recall
**AGE**	7	0	100.00%
**DEVICE**	12	0	100.00%
**MEDICALRECORD**	721	0	100.00%
**PATIENT**	1445	2	99.86%
**DATE**	11880	0	100.00%
**FAX**	6	0	100.00%
**PHONE**	407	0	100.00%
**ZIP**	143	0	100.00%
**USERNAME**	91	1	98.91%
**STREET**	414	2	99.52%
**LOCATION-OTHER**	12	2	85.71%
**IDNUM**	377	2	99.47%
**CITY**	338	2	99.41%
**DOCTOR**	3231	5	99.85%

Supplemental References

1 Stubbs, A., Kotfila, C. & Uzuner, O. Automated systems for the de-identification of longitudinal clinical narratives: Overview of 2014 i2b2/UTHealth shared task Track 1. *J Biomed Inform* **58 Suppl**, S11-19, doi:10.1016/j.jbi.2015.06.007 (2015).
2 Yang, H. & Garibaldi, J. M. Automatic detection of protected health information from clinic narratives. *J Biomed Inform* **58 Suppl**, S30-38, doi:10.1016/j.jbi.2015.06.015 (2015).
3 Aberdeen, J. *et al.* The MITRE Identification Scrubber Toolkit: design, training, and assessment. *Int J Med Inform* **79**, 849-859, doi:10.1016/j.ijmedinf.2010.09.007 (2010).
4 Dernoncourt, F., Lee, J. Y., Uzuner, O. & Szolovits, P. De-identification of patient notes with recurrent neural networks. *J Am Med Inform Assoc* **24**, 596-606, doi:10.1093/jamia/ocw156 (2017).
5 Liu, Z., Tang, B., Wang, X. & Chen, Q. De-identification of clinical notes via recurrent neural network and conditional random field. *J Biomed Inform* **75S**, S34-S42, doi:10.1016/j.jbi.2017.05.023 (2017).
6 Uzuner, O., Luo, Y. & Szolovits, P. Evaluating the state-of-the-art in automatic de-identification. *J Am Med Inform Assoc* **14**, 550-563, doi:10.1197/jamia.M2444 (2007).
7 Goldberger, A. L. *et al.* PhysioBank, PhysioToolkit, and PhysioNet: components of a new research resource for complex physiologic signals. *Circulation* **101**, E215-220 (2000).
8 Neamatullah, I. *et al.* Automated de-identification of free-text medical records. *BMC Med Inform Decis Mak* **8**, 32, doi:10.1186/1472-6947-8-32 (2008).
9 Stubbs, A. & Uzuner, O. Annotating longitudinal clinical narratives for de-identification: The 2014 i2b2/UTHealth corpus. *J Biomed Inform* **58 Suppl**, S20-29, doi:10.1016/j.jbi.2015.07.020 (2015).
